# Real-world evaluation of UK high hyperdiploidy profile using a large cohort of patients provided by HARMONY data platform

**DOI:** 10.1038/s41375-023-02046-0

**Published:** 2023-09-29

**Authors:** Amir Enshaei, Javier Martinez Elicegui, Esther Anguiano, Jude Gibson, Sulaiman Lawal, Mirella Ampatzidou, Michael Doubek, Adele K. Fielding, Edoardo La Sala, Elizabeth Middleton, Anita W. Rijneveld, Amin T. Turki, Martin Zimmermann, Ajay Vora, Anthony V. Moorman

**Affiliations:** 1 Wolfson Childhood Cancer Research Centre, Translation and Clinical Research Institute, Newcastle University Centre for Cancer, Newcastle Upon Tyne, UK; 2 Institute for Biomedical Research of Salamanca (IBSAL), Salamanca, Spain; 3 Aghia Sophia Children’s Hospital, Athens, Greece; 4 University Hospital, Brno, Czechia; 5 UCL Cancer Institute, London, UK; 6 Fondazione GIMEMA, Franco Mandelli onlus, Rome, Italy; 7 Department of Hematology Erasmus Medical Center, Rotterdam, Netherlands; 8 Department of Hematology and Stem Cell Transplantation, University Hospital Essen, Essen, Germany; 9 Department of Paediatric Haematology and Oncology, Hanover, Germany; 10 Department of Haematology, Great Ormond Street Hospital, London, UK

**Keywords:** Cancer epidemiology, Acute lymphocytic leukaemia

## To the Editor:

The most common genetic abnormality in childhood acute lymphoblastic leukaemia (ALL) is high hyperdiploidy (HeH), accounting for 30–35% of B-cell precursor ALL cases [1]. HeH is the non-random gain of chromosomes, resulting the modal chromosome number to be between 51 and 65 (or 67) [2–4]. Multiple studies have associated HeH with good outcome [1, 5, 6], however, the high frequency of HeH group resulted in accounting for up to 25% of all relapses in childhood ALL [7]. Therefore, identification of robust risk factors within this group is essential.

We recently developed and validated a novel risk profile for HeH in children based on the trisomic status of four chromosomes – 5, 17, 18 and 20 [8]. The profile defines two groups—a low risk (GR) group with a relapse risk <5% and a poor risk (PR) group with a relapse risk ~15% (i.e., similar to patients with intermediate risk genetics [9]. We developed and validated using consecutive UK paediatric trials – ALL97 and UKALL2003. To investigate its generalisability, we sought to evaluate the profile using a larger and more diverse patient cohort (including adults). The HARMONY alliance (http://www.harmony-alliance.eu), is a pan-European big data platform that has collected data on >115,000 patients with various haematological malignancies, including >10,000 patients with ALL. The objectives of this project were to (a) determine whether the UKALL-HeH risk profile validates in a large independent cohort comprising data from multiple clinical trials of childhood and adult ALL; (b) assess the added benefit of validating a risk profile using data from the HARMONY platform compared with a single country-specific cohort; (c) investigate the utility of the profile using real world karyotype data.

**Fig. 1:**
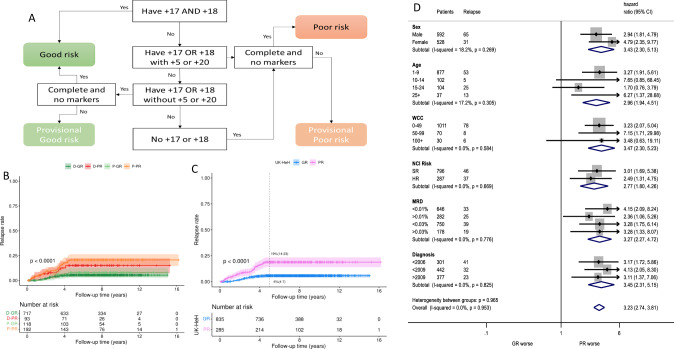
The definition and outcome of the UK-HeH profile. **A** Flow chart illustrating the rules for assigning cases to the definite good (D-GR), provisional good (P-GR), definite poor (D-PR) and provisional poor (P-PR) risk groups; (**B**) Relapse free survival for the four risk groups; (**C**) Relapse free survival of final good and poor risk groups; (**D**) Forest plot comparing the relapse rate of patients in the poor risk HeH group compared to the patients in the good risk HeH group stratified by sex, age, white cell count, NCI risk, MRD and year of diagnosis.

Our baseline cohort comprised 10,042 patients submitted to the HARMONY data platform from five data providers (including UKALL2003). All patients had a confirmed diagnosis of ALL, had been treated on a clinical trial and were aged 1–70 years old. Karyotypes with t(9;22)(q34;q11) or a chromosome pattern indicative of masked near-haploidy or masked low hypodiploidy were excluded as these cases constitute distinct subtypes. Karyotypes were assigned to four categories based on the presence of +17, +18, +5, +20 as per the recently published UKALL-HeH profile [8]. Patients with marker chromosomes (+mar) and/or incomplete karyotypes (inc) which generated uncertainty regarding classification were assigned to provisional risk groups (Fig. 1A). End of induction Minimal residual disease (MRD) was used as indicated by the data provider. All the analyses were performed in R (v. 3.6.3) [10] using survminer (v. 0.4.9) and survival (v. 3.2–7) packages. Univariate Cox regression analysis was used to estimate the risk of relapse associated with individual risk factors. Multivariate Cox regression analysis was used to build a model for predicting relapse. The fit of the final model was assessed using Harrell’s concordance index. Forest plots and the test of heterogeneity were used to examine hazard ratios across different patient subgroups or cohorts. The proportionality assumption of the models was assessed by visualising the log-log plot of survival and the Kaplan–Meier and predicted survival plots and tested using Schoenfeld residuals.

Among 6026 cases with B-cell precursor ALL who had karyotype information available, we were able to identify a cohort of 1169 karyotypes with a modal number of 51–67 chromosomes. The majority of cases (854/1169, 73%) could be confidently assigned to either the good (*n* = 746) or poor (*n* = 108) risk groups with the remaining 315 (27%) assigned to provisional risk groups: good (*n* = 119) or poor (*n* = 196) (Fig. 1A, Table S1). Comparing the demographic, clinical and outcome features of patients assigned to the provisional and definitive risk groups revealed no significant differences (Table S1, Fig. 1B, Fig. S1). This finding indicates that the UKALL-HeH profile is robust and can be applied reliably in the clinical setting even when the karyotypes have not been fully characterised. For the rest of this study, we merged the provisional cases with the relevant risk groups.

Overall, 865 (74%) cases were assigned to the UKALL-HeH good risk (GR) group and 304 (26%) to poor risk group (PR). The GR group was enriched for younger patients (*p* < 0.001) and lower white blood cell count (*p* = 0.02) (Table 1). No differences were observed for sex and MRD positivity either at 0.01% or, the HeH specific threshold of 0.03% [8]. After a median follow-up time of 8 years, the outcome of patients in the GR group was significantly superior to that experienced by patients in the PR group: EFS at 5 years 91% v 77% respectively (*p* < 0.001) (Table 1, Fig. 1B, C, Fig. S1). Cox regression analysis revealed the patients in the PR group had a three-fold increased risk of relapse, event and death compared to those patients in the GR group. Importantly, adjusting for the effect of end of induction MRD did not materially alter the hazard ratios (Table 1).

**Table 1:** Demographic, clinical and outcome features of 1169 high hyperdiploid cases stratified by UKALL HeH Risk Group.

	Total	Good risk	Poor risk	
	1169 (100)	865(100)	304(100)	*p*-value
Sex				0.31
Female	550 (47)	415 (48)	135 (44)	
Male	619 (53)	450 (52)	169 (56)	
Age				<0.001
1–9	914 (78)	706 (82)	208 (68)	
10–14	108 (9)	69 (8)	39 (13)	
15–24	106 (9)	75 (9)	31 (10)	
25+	41 (4)	15 (2)	26 (9)	
White cell count (×10^9^/L)				0.016
0–49	1026 (91)	769 (91)	257 (89)	
50–99	72 (6)	56 (7)	16 (6)	
100+	32 (3)	17 (2)	15 (5)	
End of induction Minimal Residual Disease (MRD) value				0.85
<0.01%	647 (70)	495 (69)	152 (70)	
≥0.01%	282 (30)	218 (31)	64 (30)	
HeH specific threshold				
<0.03%	751 (81)	576 (81)	175 (81)	
≥0.03%	178 (19)	137 (19)	41 (19)	
Relapse rate				
Number of relapses (%)	96 (9)	46 (6)	50 (18)	<0.001
Relapse rate at 5 years	9% (7–11)	6% (4–7)	19% (14–23)	
Unadjusted Hazard Ratio (CI), *p*-value		3.52 (2.36–5.25), *p* < 0.001		
Adjusted^a^ Hazard Ratio (CI), *p*-value		3.30 (1.97–5.52), *p* < 0.001		
Event-free survival				
Number of events (%)	145 (12)	76 (9)	69 (23)	<0.001
Event Free Survival at 5 years	87% (85–89)	91% (89–93)	77% (72–82)	
Unadjusted Hazard Ratio (CI), *p*-value		2.80 (2.02–3.87), *p* < 0.001		
Adjusted^a^ Hazard Ratio (CI), *p*-value		2.84 (1.80–4.45), *p* < 0.001		
Overall survival				
Number of deaths (%)	103 (9)	50 (6)	53 (17)	<0.001
Event Free Survival at 5 years	92% (90–94)	95% (93–96)	84% (80–88)	
Unadjusted Hazard Ratio (CI), *p*-value		3.21 (2.18–4.73), *p* < 0.001		
Adjusted^a^ Hazard Ratio (CI), *p*-value		3.72 (1.98–6.97), *p* < 0.001		

Minimal residual disease (continuous).

*CI* confidence interval.

^a^adjusted for Minimal residual disease (MRD).

Our analysis cohort was highly heterogenous comprising patients treated on paediatric and adult trials, from multiple countries and over a 26 year time period. Therefore, we performed subgroup analysis to assess whether or not the profile retained its predictive power in different patient sub-populations (Fig. S2). There was no evidence of heterogeneity in any of the subgroups examined for any of the endpoints studied: relapse (Fig. 1D), event (Fig. S2A) and death (Fig. S2B). Although the majority of patients with HeH were National Cancer Institute (NCI) standard risk (73%) our data show clearly that the UKALL HeH profile was able to identify patients with a good and poor response whose ALL was classified as NCI HR. Importantly, given that the majority of treatment protocols now use end of induction MRD to assign post-induction treatment, we demonstrate that the prognostic effect of the UKALL HeH profile was equivalent among patients with low and high MRD levels. Hence integration of MRD and genetics [11] will be crucial in future studies to ensure that all patients who have low risk HeH and all patients who have high-risk HeH are identified accurately so that appropriate therapy can be assigned.

In contrast to the original discovery and validation cohort [8] this HARMONY dataset included patients of all ages including 87 cases aged 18+ years and 41 cases 25+ year old. The patient number was more than doubled to 1169. The frequency of UKALL HeH PR increased with age (22% among 1–9 years, 37% among 10–14 years, 24% among 15–17 years, 35% among in 18–24 years and 63% among 25+ years). The prognostic impact of the UKALL HeH profile was strong among adults with patients in the PR group: hazard ratio 6.27 (1.37–28.68), *p* < 0.001) for relapse compared to patients with a GR profile (Fig. 1).

Not only did this allows us to perform robust subgroups analyses but also allowed the development of more accurate models. The 95% confidence intervals for the hazard ratios were narrower as were the standard errors of the coefficients (Table S2). As a result the prediction power (c-index) of the models for all three time points are improved (RR 6%, EFS 5% and OS 8%).

In this study, we have used data from the HARMONY alliance to perform a robust validation of a risk profile. This cohort offered several advantages. As partners in this project, we had direct and rapid access to a large amount of anonymous standardised patient data. This enabled the study to be quick because we did not have to collect data, set-up data sharing agreements or standardise the data. Thus the validation performed in this study is substantially more comprehensive compared to that previously reported [8].

Numerous studies have shown that patients with HeH have a superior outcome compared with other (non *ETV6::RUNX1*) ALL patients. However, several studies have also indicated outcome heterogeneity within this subgroup based on modal chromosome number, pattern of trisomies or the presence of structural abnormalities [12]. Our previous study demonstrated conclusively that the pattern of trisomies was the optimal method for distinguishing patients with HeH according to the risk of relapse [8]. The current analysis confirms and extends these findings.

Cytogenetic analysis is often hampered by poor quality chromosome morphology making precise chromosome identification challenging. The karyotypes collected by HARMONY span a long time-period and multiple countries; thereby representing a real-world challenge for the classifier. To account for possible misassignment of cases due to marker chromosomes and incomplete karyotypes, we classified karyotypes into definitive and provisional risk groups. There was no difference in the demographics, clinical features and outcome of patients assigned to the definitive and provisional risk groups suggesting that the classifier works well on this pan-European population.

Next, we confirmed that the risk profile validated in the overall cohort and crucially within a variety of important patient subgroups. Using the HARMONY cohort allowed us to assess the prognostic impact of the UKALL HeH risk groups in younger and older adults as well as different time periods. The increased number of cases in our study produced more accurate estimates of risk.

In conclusion, using a newly compiled cohort of ALL data made available via the HAMRONY alliance we confirmed and extended the clinical utility of the UKALL HeH risk profile.

This communication reflects the author’s view and neither IMI nor the European Union, EFPIA, or any Associated Partners are responsible for any use that may be made of the information contained therein.

### Supplementary information

Supplemental tables and figures
