# Supplementary material for: Real-world evaluation of UK high hyperdiploidy profile using a large cohort of patients provided by HARMONY data platform
Source: Leukemia. 2023 Sep 29;37(12):2493–6. doi: 10.1038/s41375-023-02046-0 (PMC10681889; doi:10.1038/s41375-023-02046-0)
Supplement: Supplementary file 1 — Supplemental tables and figures [file 41375_2023_2046_MOESM1_ESM.pdf]

Figure S1: Kaplan-Meier plots showing the event-free and overall survival of patients in the definitive (D-), provisional (P-) and final good (GR) and poor (PR) risk groups defined by the UKALL HeH profile.

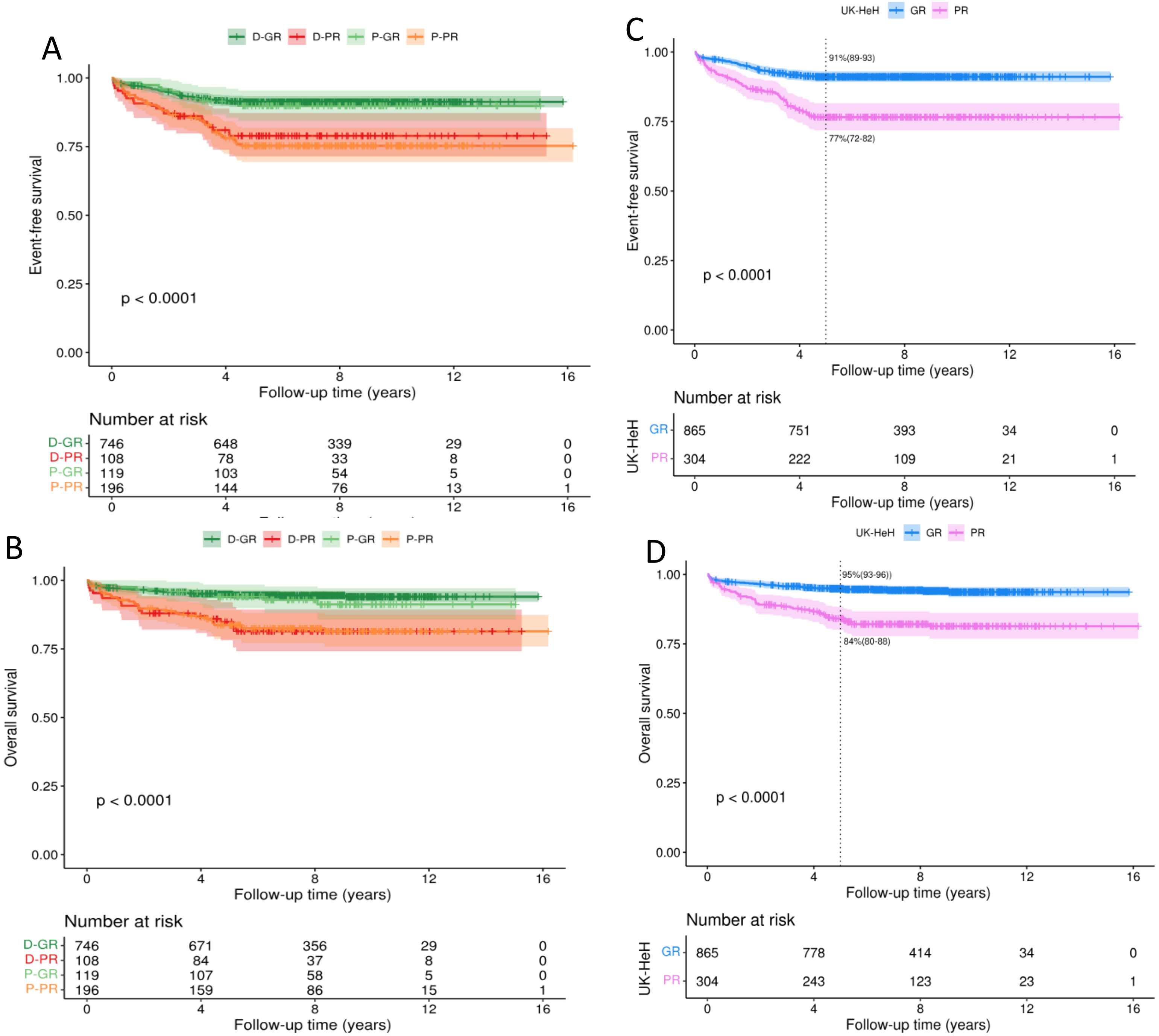

Figure S2: : Forest plots comparing the event-free (A) and overall (B) survival rate of patients in the poor risk HeH group compared to the patients in the good risk HeH group by stratified by sex, age, white cell count, NCI risk, MRD and year of diagnosis.

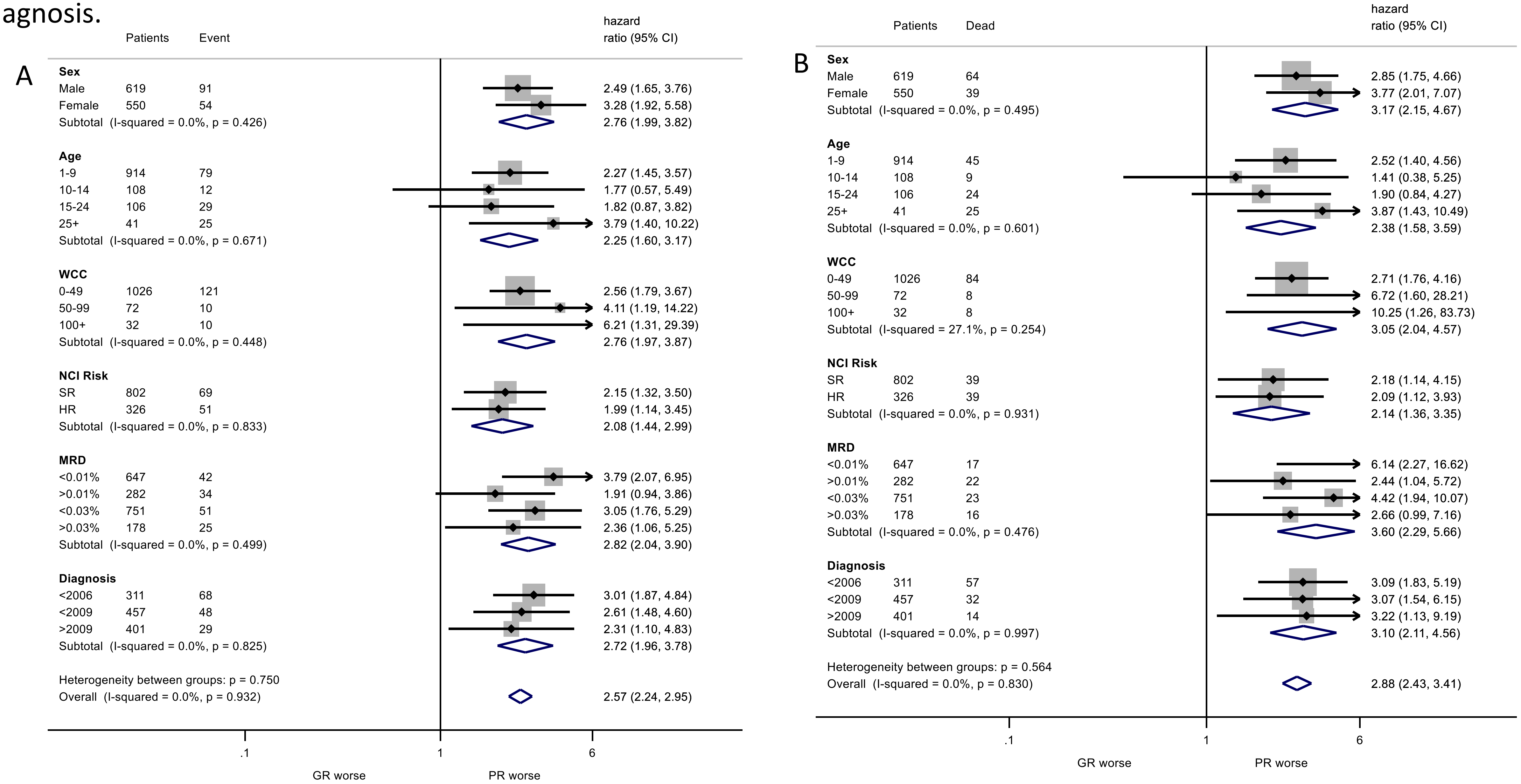

Table S1: Demographic, clinical and outcome features of High hyperdiploid cases based on provisional and definitive risk groups defined by the UKALL HeH profile

	Total	Good risk	Provisional Good risk	Poor risk	Provisional Poor risk	
	1169(100)	746(100)	119(100)	108(100)	196(100)	p-value
Sex						0.26
Female	550(47)	365(49)	50(42)	44(41)	91(46)	
Male	619(53)	381(51)	69(58)	64(59)	105(54)	
Age						<0.001
1-9	914(78)	619(83)	87(73)	79(73)	129(66)	
10-14	108(9)	55(7)	14(12)	11(10)	28(14)	
15-24	106(9)	59(8)	16(13)	9(8)	22(11)	
25+	41(4)	13(2)	2(2)	9(8)	17(9)	
White cell count ($\times 10^9$/L)						0.03
0-49	1026(91)	663(91)	106(91)	82(85)	175(91)	
50-99	72(6)	47(6)	9(8)	6(6)	10(5)	
100+	32(3)	12(2)	2(2)	8(8)	7(4)	
End of induction Minimal Residual Disease (MRD) value						0.1
<0.01%	647(70)	427(70)	68(67)	43(60)	109(76)	
>=0.01%	282(30)	184(30)	34(33)	29(40)	35(24)	
HeH specific threshold						0.06
<0.03%	751(81)	499(82)	77(75)	52(72)	123(85)	
>=0.03%	178(19)	112(18)	25(25)	20(28)	21(15)	
Relapse						
n (%)	96(9)	37(5)	9(8)	13(14)	37(19)	<0.001
Relapse rate at 5 years	9%(7-11)	95%(86-93)	92%(87-97)	85%(78-93)	79%(74-85)	
Hazard ratio (CI), p-value (comparing to the definitive group)		0.68(0.33-1.41), p=0.32		0.71(0.38-1.34), p=0.28		
Event						
n (%)	145(12)	64(9)	22(20)	12(10)	47(24)	<0.001
Event Free Survival at 5 years	87%(85-89)	91%(89-93)	90%(84-95)	79%(71-87)	75%(69-82)	
Hazard ratio (CI), p-value (comparing to the definitive group)		0.86(0.46-1.59), p=0.64		0.86(0.52-1.43), p=0.56		
Death						
n(%)	103(9)	41(5)	19(18)	9(8)	34(17)	<0.001
Overall Survival at 5 years	92%(90-94)	95%(80-93)	94%(90-98)	85%(78-92)	84%(79-89)	
Hazard ratio (CI), p-value (comparing to the definitive group)		0.73(0.36-1.51), p=0.42		1.03(0.59-1.81), p=0.91		
Abbreviations CI: confidence interval						

Table S2: Risk estimates from univariate Cox regression models and Harrel's C-index calculated using the original and HARMONY validation cohorts.

		Discovery cohort	Harmony cohort
	Number of patients	456	1169
**Relapse**	Coefficient (standard error)	0.91 (0.26)	1.26 (0.20)
	Hazard ratio (95% CI)	2.50 (1.51-4.14)	3.52(2.36-5.25)
	Harrel's concordance index	0.58	0.64
**Event**	Coefficient (standard error)	0.71 (0.25)	1.03 (0.17)
	Hazard ratio (95% CI)	2.04 (1.27-3.30)	2.80 (2.02-3.87)
	Harrel's concordance index	0.56	0.61
**Death**	Coefficient (standard error)	0.60 (0.34)	1.17 (0.20)
	Hazard ratio (95% CI)	1.82 (0.93-3.55)	3.21 (2.18-4.73)
	Harrel's concordance index	0.55	0.63

Abbreviations: CI, confidence interval;
